# A Key Silencing Histone Mark on Chromatin Is Lost When Colorectal Adenocarcinoma Cells Are Depleted of Methionine by Methionine γ-Lyase

**DOI:** 10.3389/fmolb.2021.735303

**Published:** 2021-10-01

**Authors:** Samanta Raboni, Serena Montalbano, Stephanie Stransky, Benjamin A. Garcia, Annamaria Buschini, Stefano Bettati, Simone Sidoli, Andrea Mozzarelli

**Affiliations:** ^1^ Interdepartmental Center SITEIA.PARMA, University of Parma, Parma, Italy; ^2^ Institute of Biophysics, National Research Center, Pisa, Italy; ^3^ Department of Chemistry, Life Sciences and Environmental Sustainability, University of Parma, Parma, Italy; ^4^ Department of Biochemistry, Albert Einstein College of Medicine, Bronx, NY, United States; ^5^ Department of Biochemistry and Biophysics, Epigenetics Institute, Perelman School of Medicine, University of Pennsylvania, Philadelphia, PA, United States; ^6^ Interdepartmental Centre for Molecular and Translational Oncology COMT, University of Parma, Parma, Italy; ^7^ Department of Medicine and Surgery, University of Parma, Parma, Italy; ^8^ Department of Food and Drug, University of Parma, Parma, Italy

**Keywords:** methionine, cancer therapy, histone methylation, mass spectrometry, enzyme-based therapy

## Abstract

Methionine is an essential amino acid used, beyond protein synthesis, for polyamine formation and DNA/RNA/protein methylation. Cancer cells require particularly high methionine supply for their homeostasis. A successful approach for decreasing methionine concentration is based on the systemic delivery of methionine γ-lyase (MGL), with *in vitro* and *in vivo* studies demonstrating its efficacy in cancer therapy. However, the mechanisms explaining how cancer cells suffer from the absence of methionine more significantly than non-malignant cells are still unclear. We analyzed the outcome of the human colorectal adenocarcinoma cancer cell line HT29 to the exposure of MGL for up to 72 h by monitoring cell viability, proteome expression, histone post-translational modifications, and presence of spurious transcription. The rationale of this study was to verify whether reduced methionine supply would affect chromatin decondensation by changing the levels of histone methylation and therefore increasing genomic instability. MGL treatment showed a time-dependent cytotoxic effect on HT29 cancer cells, with an IC_50_ of 30 µg/ml, while Hs27 normal cells were less affected, with an IC_50_ of >460 µg/ml. Although the levels of total histone methylation were not altered, a loss of the silencing histone mark H3K9me2 was observed, as well as a decrease in H4K20me3. Since H3K9me2/3 decorate repetitive DNA elements, we proved by qRT-PCR that MGL treatment leads to an increased expression of major satellite units. Our data indicate that selected histone methylation marks may play major roles in the mechanism of methionine starvation in cancer cells, proving that MGL treatment directly impacts chromatin homeostasis.

## Introduction

**FIGURE 1:**
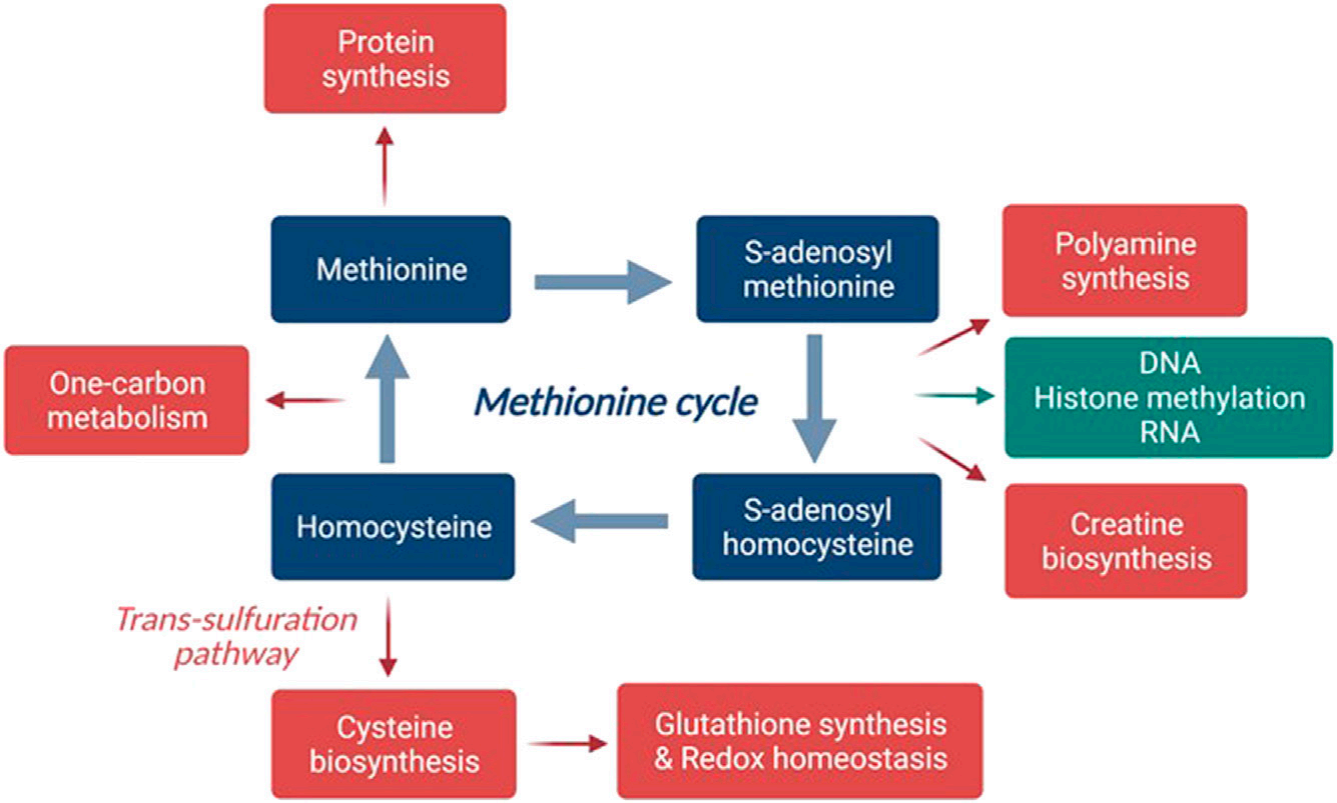
Scheme of the methionine cycle. Major pathways where methionine is being processed and utilized are shown. In green, highlight of methionine role described in this manuscript, that is, as a precursor of protein methylation. Created with BioRender.com.

A common feature of cancer cell metabolism is the ability to adapt to a frequently nutrient-poor environment to acquire the necessary supplements to both sustain high rates of cellular proliferation and build new biomass. Oncogenic mutations responsible for tumorigenesis may have an impact directly or indirectly on cellular metabolism reprogramming. In particular, deregulation in the uptake or consumption of glucose and amino acids is part of the known cancer-associated metabolic changes. The former is the Warburg effect, that is, the aerobic energy dependence on glycolysis (Warburg, 1925; Liberti and Locasale, 2016). This dependence has triggered pharmacological approaches targeting glycolytic enzymes (Scatena et al., 2008). Interestingly, cancer cells are unable to proliferate in methionine-depleted media because they are universally methionine addicted; that is, cancer cells over-use methionine for transmethylation and proliferation (Chello and Bertino, 1973; Hoffman and Erbe, 1976; Coalson et al., 1982; Stern et al., 1983; Stern et al., 1984; Hoffman, 2015). This effect has been named the “Hoffman effect” (Hoffman, 2015). The cellular requirement for methionine is manifold (Xiao and Locasale, 2021). In fact, methionine is 1) a building block amino acid for protein synthesis; 2) the precursor of S-adenosylmethionine (SAM), which plays a key role in most of the processes that involve a methyl transfer, such as DNA, RNA, and histone methylation and metabolite transformation; 3) involved in polyamine formation, in the transsulfuration pathway for cysteine and creatine biosynthesis; and 4) involved in one-carbon metabolism (Figure 1).

In malignant cells, methionine dependence does not seem to be due exclusively to the inability of synthesizing methionine, but also to changes in its metabolic use. Several hypotheses were proposed, including 1) mutations leading to a deficiency of methylthioadenosine phosphorylase, an enzyme of the methionine salvage pathway (Bigaud and Corrales, 2016); 2) changes in folate and cobalamin metabolism (Guéant et al., 2020); and 3) increased requirement for methionine, due to excess transmethylation reactions (Stern and Hoffman, 1984), not supported by the adaptive expression level of selected genes (Chaturvedi et al., 2018). A few recent studies have addressed the metabolic adaptation of normal and cancer cells to methionine deprivation (Mentch et al., 2015; Dai et al., 2018; Gao et al., 2019; Xiao and Locasale, 2021). It was also reported that methionine starvation leads cell cycle to arrest in the late S/G_2_ phase (Hoffman and Jacobsen, 1980; Guo et al., 1993), but it has not yet been established whether starvation-induced death is associated with apoptosis or other cellular processes such as autophagy.

Cancer cell dependence on methionine has triggered extensive investigations aimed at its exploitation for cancer therapy. Similarly, the treatment of acute lymphocytic leukemia with asparaginase, the asparagine-degrading enzyme, exploits the strong dependence on asparagine of cancer cells (Tallal et al., 1970; Aslanian and Kilberg, 2001), which led this enzyme to be approved by the FDA as a therapeutic agent (Cioni et al., 2021). Diets low in methionine or in combination with low intake of glucose have been applied for reduction of tumor growth, obtaining significant results in the mouse model (Breillout et al., 1987; Cavuoto and Fenech, 2012; Gao et al., 2019). An alternative strategy for decreasing methionine concentration in biological fluids, thus reducing its supply to cancer cells, is an enzyme-based supplementation therapy (Hoffman, 2015; Cioni et al., 2021). This is achieved by the systemic delivery, and, recently, by the oral administration (Kawaguchi et al., 2018a), of methionine γ-lyase (MGL), a pyridoxal 5'-phosphate (PLP) dependent enzyme that irreversibly and quantitatively degrades methionine to ammonia and methanethiol (Mamaeva et al., 2005; Manukhov et al., 2005; Ronda et al., 2011). MGL is not present in humans and other mammals, but only in bacteria, parasitic protozoa, and plants. For therapeutic development, MGL is produced by heterologous expression in *Escherichia coli* (Tan et al., 2010; Raboni et al., 2018). Studies have robustly shown MGL cytotoxic effects in cancer cell cultures, but not in normal cell lines (Morozova et al., 2018; Raboni et al., 2018). These effects are independent of whether MGL is free in solution (Morozova et al., 2013; Raboni et al., 2018), modified by conjugation with polyethylene glycol (PEG) (Sun et al., 2003; Takakura et al., 2006), encapsulated in different matrices (Hoffman, 2015; Xin et al., 2015; Morozova et al., 2018), loaded in erythrocytes (Gay et al., 2017), or delivered via gene therapy (Miki et al., 2000). Recent studies also demonstrated the therapeutic efficacy of MGL against different patient-derived orthotropic xenograft mouse models of recalcitrant cancers (Murakami et al., 2017; Kawaguchi et al., 2018b; Kawaguchi et al., 2019) and showed promise for treating patients with advanced prostate cancer (Han et al., 2020; Han and Hoffman, 2021a; Han and Hoffman, 2021b).

Importantly, methionine is the precursor of SAM, which is the methyl donor for DNA, RNA, and protein methylation, including histones. Chromatin is mainly composed of DNA and histone proteins (He et al., 2019). Methylation, together with acetylation, is by far the most abundant post-translational modification (PTM) on histones (Stransky et al., 2020). Histone methylation has complex biological roles, as it varies in its precise localization on the histone sequences and its co-existence with other PTMs (Sidoli and Garcia, 2017). When localized on the lysine 9 of histone H3 (H3K9me), it is mainly involved in forming condensed silenced heterochromatin (Xiao and Locasale, 2021). Specifically, Lamina-Associated Domains, or LADs, are heterochromatic domains sequestered at the nuclear periphery heavily decorated by H3K9me2 (Poleshko et al., 2019). On the other hand, H3K9me3 is recognized by the Heterochromatin Protein 1 (HP1) and decorates regions of repetitive DNA units including the centromere and telomere (Nicetto et al., 2019). Interestingly, previous reports documented that cell starvation in the methionine-free medium was accompanied by changes in histone methylation marks, although they were mainly focused on H3K4me3, which occupies active promoters (Mentch et al., 2015; Tang et al., 2015). Recently, a few studies reported the decrease of H3K4me3, H3K9me3, and H3K9me2 levels upon enzyme-mediated methionine deprivation in cancer cells, but not in normal cells, evaluated by immunoblotting (Machover et al., 2019; Yamamoto et al., 2020).

In this work, we exploited the analytical power of mass spectrometry to analyze for the first time the histone PTMs and the proteome profile of cancer cells upon methionine depletion caused by exposure to MGL. Mass spectrometry analyses were accompanied by phenotypic assays, evaluating cell viability and metabolic activity. We demonstrated that the presence of MGL in culture does not significantly affect the overall degree of histone methylation. However, MGL significantly impacts H3K9me2 and H4K20me2 in HT29 cells. We demonstrated that this reduction leads to an increased expression of major satellite units, indicating a reduced ability of these cancer cells to maintain heterochromatin in domains of DNA repetitive elements. Altogether, our work demonstrates that MGL affects chromatin homeostasis and represents therefore an interesting alternative or complement treatment in inducing cancer cell death in epigenetics therapy.

## Methods

### Reagents

Pyridoxal 5'-phosphate (PLP) was purchased from Sigma-Aldrich (Saint Louis, MO). The plasmid containing D-2-hydroxyisocaproate dehydrogenase (HO-Hxo-DH) gene was a kind gift of Dr. K. Muratore (University of California, Berkeley, Department of Molecular and Cell Biology, Berkeley, CA, United States).

### Methionine γ-Lyase Expression and Purification

*E. coli* BL21 (DE3) cells containing the plasmid with the gene of MGL from *C. freundii* were grown and the enzyme was purified according to the protocol previously described (Manukhov et al., 2006; Morozova et al., 2018). Protein concentration was determined by the absorbance at 280 nm (ε^0.1%^ at 280 nm = 0.8) (Morozova et al., 2010). Protein purity was above 90% as assessed by SDS-PAGE. MGL catalytic activity was determined using L-methionine as a substrate and measuring the rate of α-ketobutyrate production in the coupled reaction with HO-HxoDH monitoring the decrease of NADH absorption at 340 nm (Δε = 6220 M^−1^ cm^−1^) at 37°C. One unit of enzyme activity is defined as the amount of the enzyme that catalyzes the formation of 1.0 μmol min^−1^ of α-ketobutyrate at pH 8.0, 37°C. For cell experiments, MGL solutions were equilibrated with Phosphate Buffered Saline (PBS), pH 7.4, sterilized through Millipore GV 0.22 µm filters, and stored at −80°C.

### Cell Lines and Culture Conditions

Colorectal adenocarcinoma cell line HT29 (ATCC, HTB-38) and human skin fibroblast cell line Hs27 (ATCC, CRL1634) were obtained from ATCC (Manassas, VA, United States). Cells were cultured *in vitro* in DMEM medium (Gibco™, Thermo Fisher Scientific, Waltham, MA, United States), supplemented with 10% (v/v) fetal bovine serum (FBS) (Euroclone, Italy), 1% penicillin (100 U/ml)/streptomycin (100 μg/ml) (Euroclone, Italy), and 1% L-glutamine (2 mM) (Euroclone, Italy) at 37°C with 5% CO_2_. Cell cultures were refreshed every 2–3 days during sub-culturing. Hs27 cells were used between passage numbers 3 and 25.

### Evaluation of *In Vitro* Methionine γ-Lyase Cytotoxicity on HT29 and Hs27 Cell Lines by MTS Assay

The cytotoxic activity of MGL was evaluated on HT29 and Hs27 cell lines. In the exponential growth phase, cells were seeded at 5 × 10^4^/ml into 96-well flat-bottom microplates. Cells were cultured in DMEM supplemented with 5% FBS, 1% L-glutamine, and 1% penicillin/streptomycin, at 37°C, 5% CO_2_, in the absence of phenol red. After 24 h from seeding, different concentrations of MGL in the range 0.0005–0.5 mg/ml in a sterile PBS solution containing 100 μM PLP, pH 7.4, were added to DMEM medium supplemented with 5% FBS and 0.5 mM PLP in PBS, and co-incubated for 24, 48, and 72 h. An equal volume of a solution of 0.5 mM PLP in PBS was added to the DMEM medium in the negative control wells. The anti-proliferative activity was evaluated by a colorimetric assay (CellTiter96® Aqueous One Solution Cell Proliferation Assay, Promega Corporation, Madison, WI, United States). 20 µL of CellTiter96® AQueous One Solution Cell Proliferation Assay was added directly to culture wells and incubated for 4 h, and the absorbance was recorded at 485 nm with a 96-well plate reader (TECAN SpectraFluor Plus, Männedorf, Switzerland). Cytotoxicity experiments were carried out in quadruplicate. The IC_50_ value, that is, MGL concentration causing 50% reduction in the cell number in comparison to control cells, was calculated following the US National Cancer Institute (NCI) 60 anticancer drug screen guidelines (Shoemaker, 2006).

The methionine concentration in the medium was quantified to be 400 μM by the above-described coupled activity assay with HO-HxoDH. This concentration corresponds to half of the K_M_ calculated for MGL substrate in the standard assay conditions (Raboni et al., 2018). The consumption of methionine by MGL is completed in less than 1 h when the enzyme is present at IC_50_. As MGL reaction is irreversible and quantitative, this implies that no methionine is present in the culture medium after 1 h.

In order to verify morphological changes and cell proliferation, *in vitro* real-time microscopy observation was performed using a JuLI Smart fluorescent cell analyzer instrument (Digital Bio Technology, Boston, United States). Bright-field images were taken of living cells after different treatment times (24, 48, 72 h).

### Evaluation of *In Vitro* Methionine γ-Lyase Cytotoxicity on HT29 Cell Line by MTS Assay and Trypan Blue Exclusion Assay

HT29 cells were treated with MGL at IC_50_ (0.03 ± 0.001 mg/ml, *Results* Section) and grown in DMEM supplemented with 5% FBS, 1% L-glutamine, and 1% penicillin/streptomycin, at 37°C, 5% CO_2_, for 24, 48, and 72 h. An equal volume of a solution of 0.5 mM PLP in PBS was added to the DMEM medium in control wells. The anti-proliferative activity was evaluated by MTS assay, as above described. The MTS tetrazolium compound is reduced by cells into a colored formazan product that is soluble in the culture medium. This conversion is accomplished by NADPH or NADH produced by the dehydrogenase enzyme in metabolically active cells. Importantly, the test does not evaluate the ATP concentration after cell treatment but is used to assess cell proliferation.

HT29 cells, treated with different concentrations of MGL, as described above, for 24, 48, and 72 h, were collected by centrifugation. The Trypan blue exclusion test was used to determine the number of viable cells after treatment time. Cells were resuspended in complete medium and 0.4% Trypan blue solution (Gibco™, Thermo Fisher Scientific, Waltham, MA, United States) was added (ratio 1:1). 100 cells for each experimental condition were counted manually using a hemocytometer.

### Real-Time qRT-PCR

2 × 10^6^ cells were seeded in 25 cm^2^ flasks with 5 ml DMEM complete medium. After 24 h, cells were treated with MGL (0.03 mg/ml, see Results Section) for 72 h. As negative control, cells were incubated with 0.5 mM PLP in PBS.

After treatment, total RNA was extracted using GeneJET RNA Purification Kit (Thermo Fisher Scientific, Waltham, MA, United States) according to manufacturer’s protocol. The RNA concentration was measured determining the A260/A280 ratio using a NanoDrop 2000 Spectrophotometer (Thermo Fisher Scientific, Waltham, MA, United States). 1 µg of total RNA sample was reverse-transcribed using QuantiTect^®^ Reverse Transcription Kit (Qiagen, Hilden, Germany) according to manufacturer’s protocol. The complementary DNA (cDNA) samples were used as templates of qRT-PCR reactions carried out with QuantStudio™ 3 Real-Time PCR System (Thermo Fisher Scientific, Waltham, MA, United States) and QuantiNova™ SYBR^®^ Green PCR Kit (Qiagen, Hilden, Germany). Three technical replicates were used to validate the amplification specificity. Target gene expression was normalized to the expression of GAPDH gene. GAPDH was selected as internal control after assessing that there were no changes in expression in untreated versus treated cells. The comparative Ct method was used for relative mRNA quantification. Amplification conditions were 95°C for 2’ to PCR initial heat inactivation, followed by 40 cycles at 95°C for 5’’ and 60°C for 10’’.

Primer sequences selected for qPCR are in the table in the next page.

**Table:** 

Primers		
—	Forward	Reverse
GAPDH	AAC​TTT​GGC​ATT​GTG​GAA​GG	CAC​ATT​GGG​GGT​AGG​AAC​AC
Major satellite	TGG​AAT​ATG​GCG​AGA​AAA​CTG	AGG​TCC​TTC​AGT​GGG​CAT​TT

### Protein Extraction and Digestion

Cells were seeded at 2.5 × 10^5^/well into 12-well flat-bottom microplates. Cells were cultured in DMEM supplemented with 5% FBS, 1% L-glutamine, and 1% penicillin/streptomycin, at 37°C, 5% CO_2_, in the absence of phenol red. Treated cells were rinsed twice with PBS and subjected to trypsin treatment to detach cells from the plate. Cells were centrifuged at 151 g for 10 min at 4°C to remove supernatant. The cell pellet was rinsed twice with PBS, centrifuged at 1,500 rpm for 10 min at 4°C, and resuspended in 100 µL of ice-cold lysis buffer (50 ammonium bicarbonate, pH 8.5, 1% (w/v) sodium deoxycholate (SDC), and protease inhibitor cocktail (Complete™ ULTRA Tablets, Roche)). The suspension was incubated for 30 min on ice and sonicated at high power in Bioruptor® (Diagenode, Denville, NJ, United States) for 10 cycles (sonication cycle: 30 s ON, 30 s OFF) at 4°C. The resulting mixture was centrifuged at 12,000 g for 15 min at 4°C. Total protein content was determined by BCA assay without any interference from SDC. Samples were incubated for 60 min with 10 mM DTT at room temperature and alkylated with 20 mM iodoacetamide for 45 min at room temperature in the dark. Prior to in-solution enzymatic digestion with trypsin overnight at 25°C (1:50 trypsin:protein ratio), sample solutions were evaporated to half of the starting volume by vacuum centrifugation to increase protein concentration maintaining SDC at a concentration tolerable for trypsin activity. Acidic inactivation of trypsin with TFA allowed the acidic precipitation and removal of SDC. Samples were centrifuged for 15 min at 4°C, 13,000 rpm to collect the supernatant.

### Histone Extraction and Digestion

2.5 × 10^5^ cells were seeded in 12-well flat-bottom microplates, with 2 ml of DMEM complete medium. After treatment, HT29 and Hs27 cell nuclei were isolated and histone proteins were extracted according to Sidoli et al. (2016), with minor modifications. Briefly, nuclei were isolated by using the Nuclei Isolation Buffer (NIB) (Sidoli et al., 2016). The first round of NIB treatment was performed by using NIB +0.2% NP-40 at a volume ratio of 9:1 buffer:cell pellet to lyse the cell membrane, and two subsequent washes without NP-40 to remove detergents. Each step was followed by centrifugation at 600 g to collect the pellet. Histones were acid-extracted from nuclei with chilled 0.2 M H_2_SO_4_ and incubated for 2 h at 4°C, followed by precipitation with 33% trichloroacetic acid (TCA) overnight at 4°C. Then, the supernatant was removed and the tubes were rinsed with ice-cold acetone containing 0.1% HCl, centrifuged, and rinsed again with 100% ice-cold acetone. After the final centrifugation, the supernatant was discarded and the pellet was dried using a vacuum centrifuge.

Derivatization and digestion were performed using the propionylation protocol (Garcia et al., 2007). Histones were dissolved in 30 μL of 50 mM ammonium bicarbonate, pH 8.0. A solution containing propionic anhydride mixed with acetonitrile in a ratio of 1:3 (v/v) was added to histone solutions in the ratio of 1:4 (v/v) for 20 min, at room temperature. Two rounds of propionylation were performed to ensure complete derivatization. Histones were then digested with 1 µg of trypsin diluted in 50 mM ammonium bicarbonate (1:20, enzyme:sample) overnight at room temperature. The derivatization reaction was repeated twice to process peptide N-termini. Samples were dried in a vacuum centrifuge.

### LC−MS/MS Acquisition

Prior to mass spectrometry analysis, samples were desalted using C18 Stage-tips. Stage-tips were manufactured in-house by sealing a disk of C_18_ material (Empore) at the bottom of a P200 tip. Samples were then resuspended in 10 µL of 0.1% TFA and loaded onto a Dionex RSLC Ultimate 300 (Thermo Scientific), coupled online with an Orbitrap Fusion Lumos (Thermo Scientific). Chromatographic separation was performed with a two-column system, consisting of a C-18 trap cartridge (300 µm ID, 5 mm length) and a picofrit analytical column (75 µm ID, 25 cm length) packed in-house with reversed-phase Repro-Sil Pur C18-AQ 3 µm resin. To analyze the proteome, peptides were separated using a 180 min gradient from 4 to 30% buffer B (buffer A = 0.1% formic acid; buffer B = 80% acetonitrile +0.1% formic acid) at a flow rate of 300 nl/min. The mass spectrometer was set to acquire spectra in a data-dependent acquisition (DDA) mode. Briefly, the full MS scan was set to 300–1,200 m*/z* in the Orbitrap with a resolution of 120,000 (at 200 m*/z*) and an AGC target of 5 × 10^5^. MS/MS was performed in the ion trap using the top speed mode (2 s), an AGC target of 10^4,^ and an HCD collision energy of 35.

To analyze the histones, peptides were separated using a 60 min gradient from 4 to 30% buffer B at a flow rate of 300 nl/min. The mass spectrometer was set to acquire spectra in a data-independent acquisition (DIA) mode. Briefly, the full MS scan was performed acquiring the *m/z* range 300–1,100 in the Orbitrap with a resolution of 120,000. MS/MS was performed in the Orbitrap with consecutive isolation windows of 50 m*/z* with an AGC target of 2 × 10^5^ and an HCD collision energy of 30.

### Proteomics and Histone Data Analysis

Proteome raw files were searched with Proteome Discoverer software (v2.4, Thermo Scientific) using SEQUEST as a search engine and the SwissProt human database. The search for total proteome included variable modifications of N-terminal acetylation and fixed modification of carbamidomethyl cysteine. Trypsin was specified as the digestive enzyme with two missed cleavages allowed. Mass tolerance was set to 10 ppm for precursor ions and to 0.2 Da for product ions. Peptide and protein false discovery rate was set to 1%. Prior to statistics, proteins were log2 transformed, normalized by the average value of each sample, and missing values were imputed, using a normal distribution, 2 standard deviations lower than the mean.

Histone peptides raw files were processed using EpiProfile 2.0 software (Yuan et al., 2018). From the extracted ion chromatogram, the area under the curve was obtained and used to estimate the abundance of each peptide. Selected chromatograms of low abundance peptides were manually inspected to verify the accuracy of the signal extraction (example in Supplementary Figure S5). In order to achieve the relative abundance of post-translational modifications (PTMs), the sum of all different modified forms of a histone peptide was considered as 100% and the area of the particular peptide was divided by the total area for that histone peptide in all of its modified forms. The relative ratio of two isobaric forms was estimated by averaging the ratio for each fragment ion with a different mass between the two species. The resulting peptide lists generated by EpiProfile were exported to Microsoft Excel and further processed for a detailed analysis.

### Statistical Analysis

For cell count and proliferation analysis, a two-way ANOVA was used to determine significant differences among treatments. Bonferroni *post hoc* test was used for pairwise comparisons. Statistical tests were performed using SPSS 25.0 software (IBM Corp. 2017). For proteomics and histone analysis, statistical regulation was assessed using a heteroscedastic *t*-test (if *p*-value < 0.05). Data distribution was assumed to be normal, but this was not formally tested.

## Results

### Methionine γ-Lyase Cytotoxicity Against Colorectal Adenocarcinoma HT29 Cell Line

**FIGURE 2:**
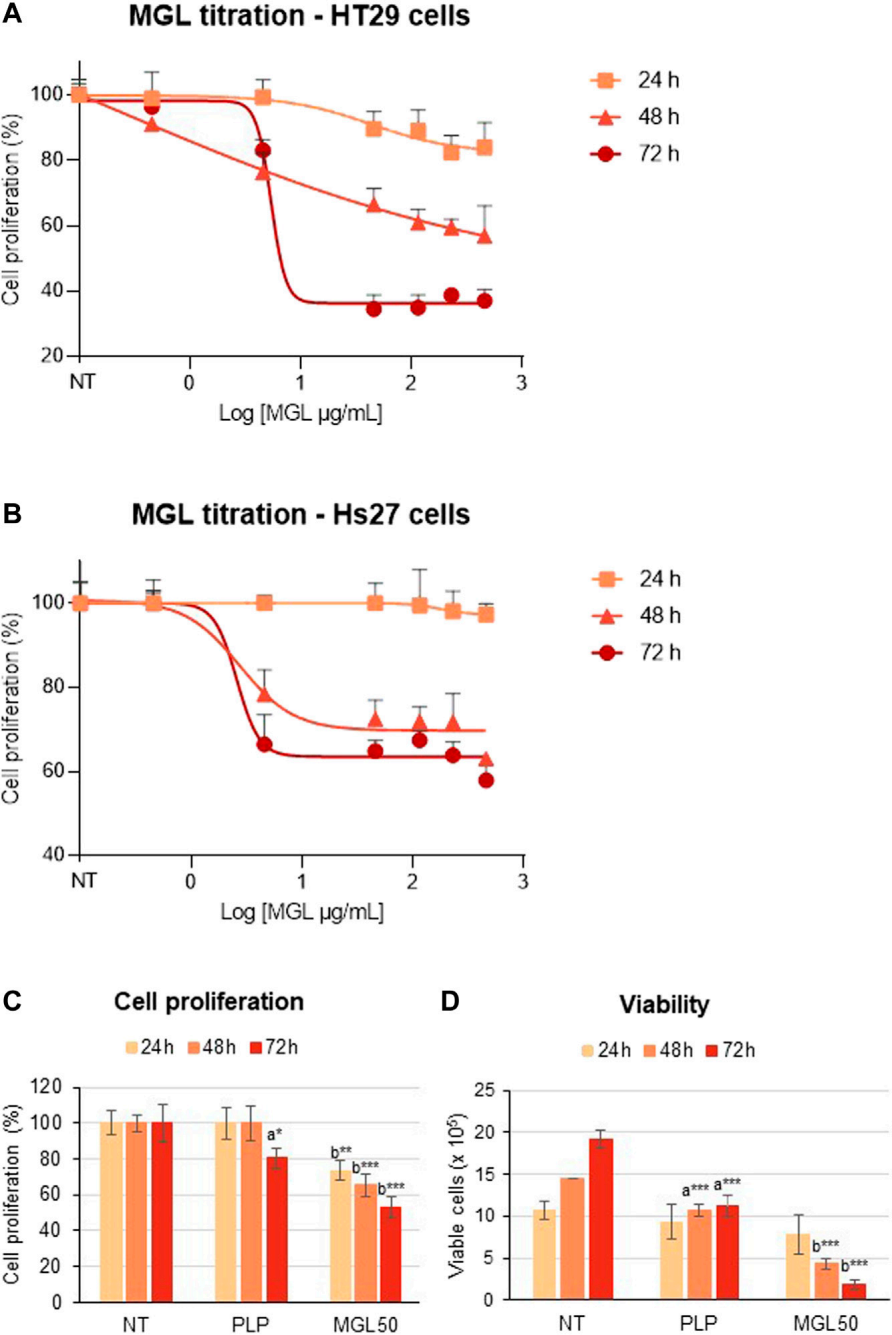
MGL cytotoxicity against colorectal adenocarcinoma HT29 cell line and normal Hs27 human fibroblast cell line. **(A)** Dependence of HT29 cancer cell proliferation as a function of MGL concentrations at 24, 48, and 72 h of incubation evaluated using the MTS Assay. Cells were seeded (5 × 10^4^ cells/mL) into 96-well plates, and after 24 h, they were treated with MGL. Non-treated (NT) cells and cells treated with control vehicle (0.5 mM PLP in PBS) did not show any change up to 72 h. Data analysis showed an IC_50_ for MGL of 0.030 ± 0.001 mg/ml for HT29 cells incubated for 72 h. ANOVA, Bonferroni post hoc test, * = *p* ≤ 0.05; ** = *p* ≤ 0.01; and *** = *p* ≤ 0.001. **(B)** Dependence of Hs27 normal fibroblast cell proliferation as a function of MGL concentrations at 24, 48, and 72 h of incubation. Cells were seeded (5 × 10^4^ cells/mL) into 96-well plates, and after 24 h, they were treated with MGL. Non-treated (NT) cells and cells treated with control vehicle (0.5 mM PLP in PBS) did not show any change up to 72 h. Data analysis showed an IC_50_ for MGL >0.46 mg/ml, at all incubation times. ANOVA, Bonferroni post hoc test, * = *p* ≤ 0.05; ** = *p* ≤ 0.01; and *** = *p* ≤ 0.001. **(C)** HT29 cells were seeded (5 × 10^4^ cells/mL) into 96-well plates, and after 24 h, they were treated with 0.030 mg/ml MGL (MGL50, concentration of MGL50 able to reduce cell viability to 50%). After 24, 48, or 72 h treatment, the cell proliferation was evaluated using the MTS Assay. (**D)** The Trypan Blue exclusion test was used to determine the number of HT29 viable cells after treatment. Data are represented as means ± SD. NT, non-treated; PLP, control vehicle. Statistical analysis was made, at each incubation time, between NT and PLP **(A)** and between PLP and MGL **(B)** reporting significant differences. ANOVA, Bonferroni post hoc test, * = *p* ≤ 0.05; ** = *p* ≤ 0.01; and *** = *p* ≤ 0.001.

We analyzed the cytotoxic properties of MGL on HT29 colorectal adenocarcinoma cell line. Cells were incubated with different concentrations of MGL (0.00046–0.46 mg/ml) and cell proliferation was assessed upon 24, 48, and 72 h of incubation (Figure 2). A concentration-dependent decrease in cell proliferation was observed at each incubation time (Figure 2A)**.** The concentration that reduces HT29 cell proliferation to 50% (IC_50_, MGL50) was calculated to be 0.0030 ± 0.001 mg/ml at 72 h (Figure 2A), in good agreement with previous studies [e.g., (Raboni et al., 2018)]. When HT29 cells were directly exposed to MGL50 for 24–72 h, a constant decrease in cell proliferation and cell viability was observed (Figures 2C,D, Supplementary Figure S1). More specifically, the proliferation of MGL-treated HT29 cells is strongly inhibited at 72 h, with the number of viable cells lowered to 25% of the initial value. Concomitantly, the effect on the metabolic activity, measured by the mitochondrial activity, is 75% at 24 h and 50% at 72 h compared to non-treated (NT) control.

In contrast, Hs27 normal human fibroblast cell line was much less affected by MGL treatment (Figure 2B), showing an IC_50_ > 0.46 mg/ml. These findings indicate that HT29 cells, as many other cancer cell lines according to the literature, are highly susceptible to the effects of MGL, that is, to the depletion of methionine, more than non-malignant cells. A minimal but significant decrease in viability caused by the PLP on HT29 cells should also be noted. These data are in line with previous results that showed increased cytotoxicity of 5-fluorouracil induced by PLP on the HT29 cell line (Machover et al., 2018).

### Effects of Methionine γ-Lyase Treatment on Histone Methylation and Acetylation

**FIGURE 3:**
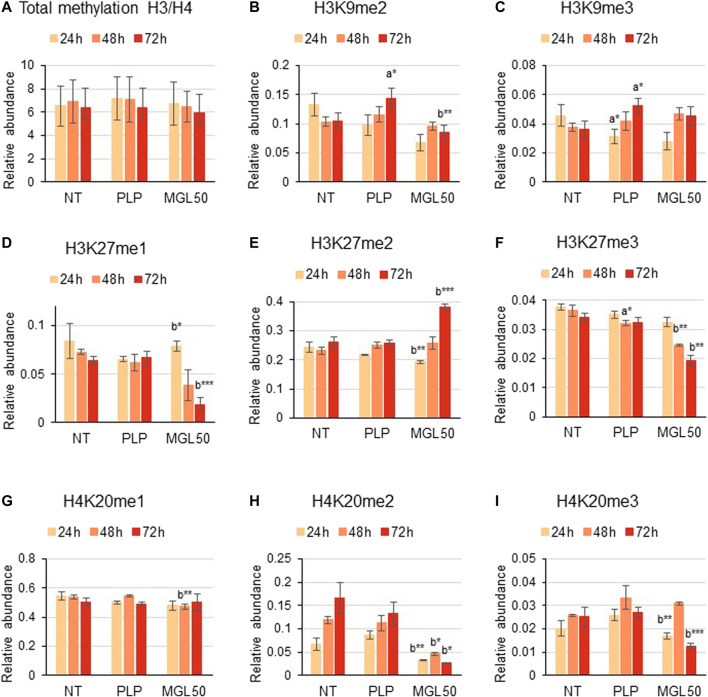
Effects of MGL50 on histone methylation of HT29 cells. HT29 cells were seeded (2.5 × 10^5^ cells/well) into 12-well plates and then treated with 0.03 mg/ml MGL (MGL50) for 24, 48, and 72 h. Cells were collected, histones were extracted, and peptides were analyzed by mass spectrometry. **(A)** Total methylation levels of H3 plus H4, and relative abundance of the marks **(B)** H3K9me2, **(C)** H3K9me3, **(D)** H3K27me1, **(E)** H3K27me2, **(F)** H3K27me3, **(G)** H4K20me1, **(H)** H4K20me2 and **(I)** H4K20me3. Data are represented as means ± SD. NT, non-treated; PLP, control vehicle; MGL50, concentration of MGL able to reduce cell viability to 50%. Statistical analysis was carried out comparing PLP vs. NT **(A)** and MGL50 vs. PLP. **(B)** ANOVA, Bonferroni post hoc test, * = *p* ≤ 0.05; ** = *p* ≤ 0.01; and *** = *p* ≤ 0.001.

We then investigated the effect of methionine depletion caused by MGL treatment at the molecular level*.* Specifically, we evaluated whether methionine depletion affects histone post-translational modifications, that is, methylation, acetylation, and phosphorylation. Interestingly, incubation of HT29 cells with MGL50 for 24, 48, or 72 h did not alter the total methylation levels of histone 3 (H3) and histone 4 (H4) (Figure 3A) or modifications with a very slow turnover (Sidoli et al., 2019) like the constitutive heterochromatic mark H3K9me3 (Figure 3C). However, methylation marks such as H3K9me2, H3K27me1, H3K27me3, H4K20me2, and H4K20me3 showed substantially decreased levels (Figures 3B,D,F,H,I, respectively), whereas the marks H3K27me2 and H4K20me1 showed a very modest increase after MGL50 treatment (Figures 3E,G, respectively). Interestingly, H4K20me2 showed a partial increase in the untreated samples from 24 to 72 h (Figure 3H). H4K20me2 is a modification regulated during the cell cycle and during DNA repair (Paquin and Howlett, 2018; Simonetta et al., 2018), suggesting that it might accumulate in association with cell cycle progression of actively dividing cells, such as the untreated ones. However, it is important to mention that neither the overall methylation levels nor the methylated marks of Hs27 cells were affected by MGL treatment (Supplementary Figure S2).

**FIGURE 4:**
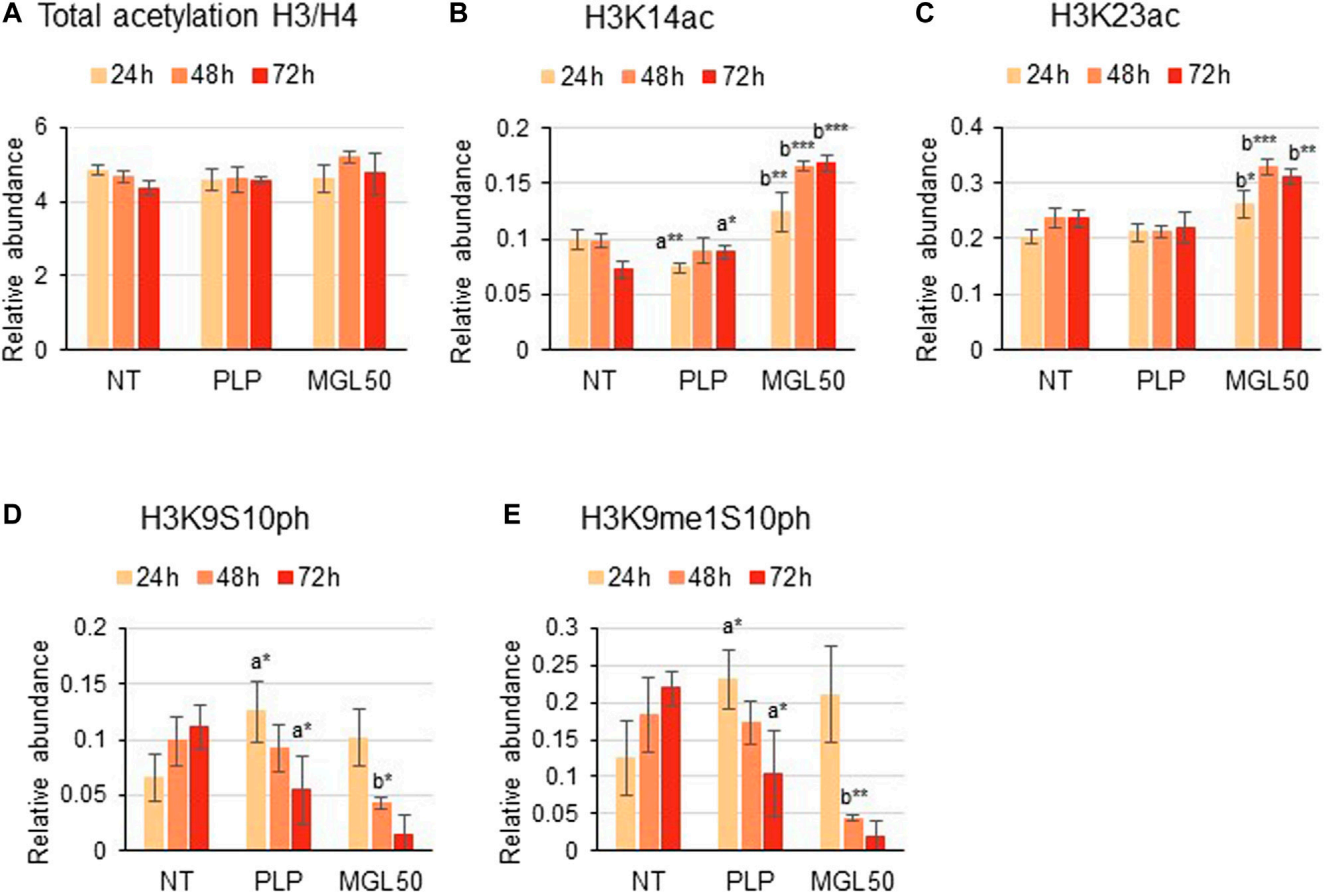
Effects of MGL50 on histone acetylation and phosphorylation. HT29 cells were seeded (2.5 × 10^5^ cells/well) into 12-well plates and then treated with 0.03 mg/ml MGL (MGL50) for 24, 48, and 72 h. Cells were collected, the histones were extracted and the peptides were analyzed by mass spectrometry. **(A)** Total acetylation levels of H3 plus H4 and relative abundance of the marks **(B)** H3K14ac, **(C)** H3K23ac, **(D)** H3K9S10ph, **(E)** H3K9me1S10ph. Data are represented as means ± SD. NT, non-treated; PLP, control vehicle; MGL50, concentration of MGL50 able to reduce cell viability to 50%. Statistical analysis was carried out comparing PLP vs. NT **(A)** and MGL50 vs. PLP. **(B)** ANOVA, Bonferroni post hoc test, * = *p* ≤ 0.05; ** = *p* ≤ 0.01; and *** = *p* ≤ 0.001.

Furthermore, HT29 cells treated with MGL50 for 24, 48, or 72 h showed conserved H3 and H4 total acetylation levels (Figure 4A), similar to the levels observed for Hs27 cells (Supplementary Figure S3). However, specific acetylation marks such as H3K14ac and H3K23ac significantly increased on both cell lines (Figures 4B,C, Supplementary Figure S3). Phosphorylation marks such as H3K9S10ph and H3K9me1S10ph were also significantly affected by MGL50 treatment (Figures 4D,E **,** respectively). Overall, these findings demonstrate that methionine depletion caused by MGL on HT29 cells is followed by changes in selected histone methylation, acetylation, and phosphorylation marks.

### Effects of Methionine γ-Lyase Treatment on the Proteome of HT29 Cells

**FIGURE 5:**
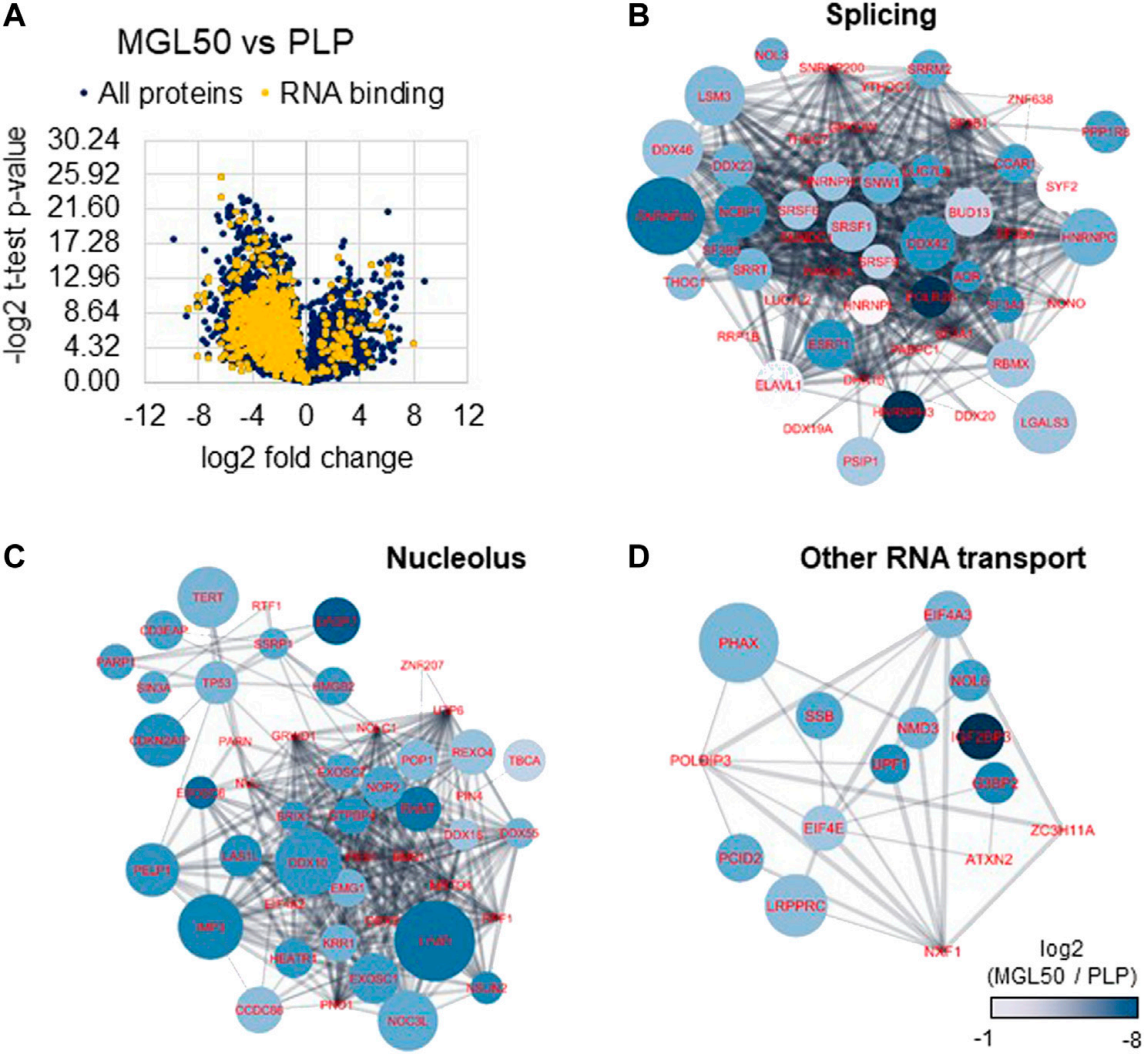
Proteome regulation of MGL vs. PLP conditions in HT29 cancer cell**s**. **(A)** Volcano plot represents the MGL50 vs. PLP control fold change and *p*-value. Proteins highlighted in yellow are proteins involved in RNA binding as molecular function (Gene Ontology). **(B)** Network representation of proteins enriched in the PLP control, that is, depleted by MGL treatment, involved in RNA splicing, **(C)** nucleolus, and **(D)** other RNA transport functions. The network was generated using Cytoscape (Shannon et al., 2003) and protein nodes were connected using the STRING App (Doncheva et al., 2019). Nodes color represents the log2 fold change of the protein abundance in MGL vs. PLP; negative values indicate higher abundance in PLP. The node size is proportional to the −log2 of the calculated *p*-value (heteroscedastic *t*-test), that is, larger nodes indicate more significant enrichment.

MLG-treated HT29 cells were then subjected to proteomics analysis to investigate the impact of methionine depletion on protein expression. It is plausible that, to respond to methionine scarcity, either the cells would activate specific pathways or they would reduce their overall gene expression to minimize protein production and potentially spurious transcription. This phenomenon is not fully characterized for histone methylation, but it is known to happen with DNA methylation (Neri et al., 2017). The proteomic analysis highlighted an overall lower content of proteins in MGL50 samples compared to PLP control after 72 h treatment (Figure 5A), demonstrated by a larger distribution of proteins in the negative end of the volcano plot. By performing a Gene Ontology analysis with GOrilla (Eden et al., 2009) (also Supplementary Figure S4), we defined “RNA binding” to be the most enriched molecular function in proteins depleted by MGL treatment (Figure 5A, in yellow). In particular, we identified a significant downregulation of the majority of the spliceosome complex (Figure 5B), including the abundant U5 small nuclear ribonucleoprotein 40 kDa protein (SNRNP40). Proteins annotated as nucleolar were also found to be enriched in the control, that is, less abundant during MGL treatment (Figure 5C). In this pool, we identified members of the exosome complex such as EXOSC6 and EXOSC7, suggesting that MGL-treated cells exhibit a reduced capacity of RNA degradation. Finally, we also grouped proteins with other roles associated with RNA binding, in particular proteins involved in RNA transport (Figure 5D). The most enriched protein in this regard was the Phosphorylated adapter RNA export protein (PHAX), meaning that MGL-treated cells had significant losses in proteins involved in translocating RNA from the nucleus to the translational machinery. Altogether, the proteomics analysis suggests that our original second hypothesis might be the most correct; that is, cells treated with MGL reduce their ability in transcription and therefore they minimize the production of proteins involved in transcription-to-translation. This result might also be the consequence of the reduced rate of the cell cycle of cells undergoing MGL treatment (Hoffman and Jacobsen, 1980; Guo et al., 1993).

### Analysis of Major Satellite Units Upon Methionine γ-Lyase Treatment by qRT-PCR

**FIGURE 6:**
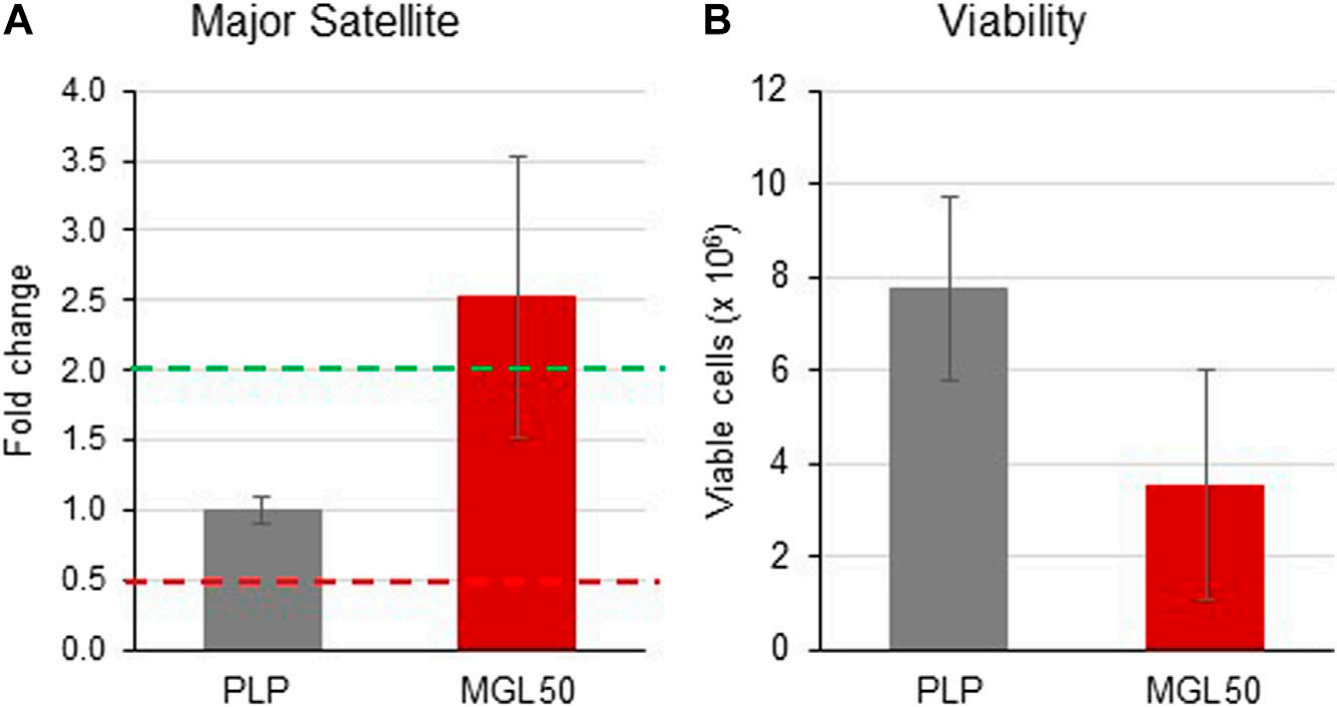
Effects of MGL in the expression of the major satellite units. HT29 cells were seeded (2 × 10^6^ cells/flask) into 25 cm^2^ flasks with complete medium and then treated with MGL50 for 72 h. **(A)** The total RNA was extracted, quantified and 1 µg was reverse-transcribed. The complementary DNA (cDNA) was used as a template of qRT-PCR reactions. Major satellite gene expression was normalized using the GAPDH gene as a housekeeping reference gene. **(B)** The Trypan Blue exclusion test was used to determine the number of viable cells after treatment. PLP, control vehicle; MGL50, concentration of MGL50 able to reduce cell viability to 50%.

We found that MGL50 treatment was able to reduce the levels of H3K9me2, a mark that is known to decorate repetitive DNA elements. We next performed a qRT-PCR to evaluate whether MGL treatment was interfering with the expression of major satellite regions (Figure 6). The 2^−ΔΔCT^ method was used to calculate relative changes in gene expression determined from qRT-PCR experiments (Wai et al., 2020). Data are presented as fold change in Major Satellite gene expression in MGL50 treated cells normalized to the internal control gene (GAPDH) and relative to the control vehicle (0.5 mM PLP).

The distribution of threshold cycle values of GAPDH gene transcription in different experiments, shown in Supplementary Figure S6 A, indicated expression stability of the GAPDH housekeeping gene, thus proving reliable qRT-PCR results.

In the present study, upregulated mRNA expression was defined by a fold change ≥2.0, “normal” expression by a fold change ranging from 0.5001 to 1.9999, and downregulated mRNA expression by a fold change ≤0.5. MGL treatment led to an increased expression of major satellite units (fold change = 2.529) compared to the control vehicle PLP (Figure 6A). In the same experiment, in conjunction with the altered transcription induced by treatment with MGL50, we also observed, as expected from previous experiments (Figure 2), a reduction of viable HT-29 cells (Figure 6B). These findings suggest a reduced ability of HT29 cells to maintain heterochromatin in domains of DNA repetitive elements in a methionine-depleted environment.

## Discussion

Our study was primarily aimed at characterizing the mechanisms by which cancer cells adapt to methionine depletion caused by the catalytic activity of MGL, a potentially powerful drug that impairs their proliferation and viability. For this reason, as we determined that MGL specifically affects cancer cells, but not normal cells, we collected proteomic and qRT-PCR data only on cancer cells, leaving to future experiments a detailed comparison of proteomic data between normal Hs27 and cancer HT29 cell lines. Here, we found that the growth inhibition of colorectal adenocarcinoma cancer cell line depleted in methionine by the enzymatic action of MGL is correlated with an alteration of methylation as well as acetylation and phosphorylation of histone 3 and 4 specific marks. In addition, a minimization in the production of proteins involved in transcription-to-translation was observed, hinting that methionine depletion triggers a cascade of adaptive cellular events. This is in line with recent publications proving that methionine depletion affects heterochromatin (Haws et al., 2020; Xiao and Locasale, 2021) and that MGL treatment in cancer cell cultures has cytotoxic effects [e.g., (Raboni et al., 2018)]. Previous studies were carried out aimed at evaluating the synergic effects of specific methionine-poor diet and anti-cancer drugs (Lien and Vander Heiden, 2019). In particular, the efficacy of a methionine-depleted diet was investigated in the presence of cisplatin (Hoshiya et al., 1996), cytotoxic agents (Poirson-Bichat et al., 2000), and 5-fluorouracyl (Gao et al., 2019). Denu and collaborators (Haws et al., 2020) demonstrated that SAM depletion in proliferating HCT116 human colorectal cancer cells decreases the levels of H3K9me2/3, activates *de novo* H3K9 mono-methylation on newly synthesized and chromatin-bound histone H3, and stimulates redistribution of H3K9me1 to repetitive and transposable genomic loci after 24 h proliferation in a methionine-depleted medium. We mostly observed a decrease in H3K9me2 abundance together with another major silencing mark H3K27me3, a modification with more rapid turnover (Zee et al., 2010) than the more stable and epigenetically inherited H3K9me3 (Audergon et al., 2015). On the contrary, H3K9me1 is known to be conserved also in the scarcity of SAM (Haws et al., 2020). This active preservation of H3K9 mono-methylation is suggested to be an adaptive mechanism to funnel scarcely available methionine and SAM toward specific chromatin modification states. This possibly maintains heterochromatin stability and safeguards epigenetic information in response to SAM perturbation that could mimic the life-experienced natural fluctuations in cells. The results of Haws et al. (2020) point out to a specific role of H3K9 mono-methylation “as an indispensable mechanism to support heterochromatin stability and global epigenetic persistence in response to SAM depletion.” Indeed, we did not observe changes of H3K9me1 in methionine depletion (72 h).

Our results are also in good agreement with those observed in closely related experiments, carried out by Hoffman and coworkers, where MGL was exploited for methionine depletion in normal and cancer cell lines (Yamamoto et al., 2020). In fact, H3K9Me3 was found to significantly decrease in the human lung cancer cell line H460 and human colon cancer cell line HCT116 and remained constant in human normal Hs27 cells.

Interestingly, MGL activity may produce ammonia as a minor byproduct. However, we have considered it to not be a direct contributor in cell viability. The concentration of ammonia in the medium produced by methionine degradation is contingent on the medium pH, which is maintained constant by PBS and CO_2_ to counterbalance the pH variations due to cell metabolism. At physiological pH of 7.2, only about 1% of the total concentration of ammonia and ammonium is present as NH3, the rest being NH4^+^. The amount of ammonium produced in the reaction catalyzed by the enzyme is stoichiometric to the amount of methionine in the medium (400 uM); considering the pH and temperature of the medium, the equilibrium between ammonium and ammonia strongly favors the former and does not approach the total concentrations of ammonia and ammonium (2-3 mM) that is reported to reduce cell growth.

Cell-intrinsic mechanisms of nutrient sensing are intimately linked to adaptive metabolic responses (Sanderson et al., 2019; Xiao and Locasale, 2021). Cells monitor available resources to coordinately tune metabolic responses to the supply of nutrients. In particular, several mechanisms are triggered in response to amino acid deprivation, cumulatively called amino acid response (Kilberg et al., 2005). Some mechanisms are amino acid specific, some are in common, but, overall, the metabolic interplay, regulatory processes, and mechanisms are far from being fully deciphered both in normal and cancer cells. It is of note the recent discovery that SAM is sensed by the SAM-sensor SAMTOR for the mTORC1 pathway and it links methionine availability and one-carbon metabolism to mTORC1 signaling (Gu et al., 2017). Unlike leucine and arginine, direct sensors upstream of mTORC1, methionine is sensed indirectly through SAM, which is a central metabolite required for most methylation reactions (Gu et al., 2017). The methyl donor SAM binds to SAMTOR, disrupts the SAMTOR-GATOR1 complex, and signals methionine sufficiency to mTORC1. Reduction of SAM levels below the dissociation constant induced by methionine restriction promotes the association of SAMTOR with GATOR1, thereby inhibiting mTORC1 signaling. In isolated rat hepatocytes, deprivation of methionine induced a greater inhibitory effect on protein synthesis than did deprivation of other essential amino acids (Everson et al., 1989). In HEK293T cells, methionine starvation was demonstrated to play a unique regulatory role in translation initiation that is not dependent upon eIF2 phosphorylation (Mazor et al., 2018). In MCF7 breast cancer cells, as well as PC3 prostate cancer cells, deprivation of most amino acids but glycine reduced cell number to approximately the same extent, whereas methionine deprivation led to the most dramatic reduction in cell number (Tang et al., 2015). The specific methionine-deprived transcriptional response required creatine biosynthesis, leading to SAM depletion and reduction in histone methylation (Tang et al., 2015). In addition, in cells in which arginine/glycine-dependent creatine biosynthesis is required for methionine-deprivation response, a crosstalk among the metabolism of arginine, glycine, and methionine was observed (Tang et al., 2015). These cumulative observations well fit with our detection of alterations in H3 and H4 marks as well as the significant decrease of RNA proteins involved in translation events. Indeed, a recent study from Deblois et al. (2020) shed light on the importance of maintaining transcriptional control over the activation of potentially detrimental transposable elements to sustain cancer progression in taxane-resistant triple-negative breast cancer. In addition, prolonged deprivation of methionine in association with cysteine was reported to reactivate in different cancer cells exogenous and endogenous integrated silenced genomic sequences (Palmisano et al., 2012; De Vito et al., 2018). Independently of mTOR and GCN2, this highly conserved reactivation response is proposed to be linked to translational block at the ribosome and ribosomal stress/dysfunction, possibly through a MAPK cascade. This pattern of repeat expression upon Met/Cys starvation preferentially upregulates endogenous retrovirus sequences and differs from that observed in mammalian senescent cells and aging somatic tissues, where non-LTR retrotransposons (LINEs, SINEs, and SVAs) were found to increase expression and ultimately transposition (De Cecco et al., 2013a; De Cecco et al., 2013b).

## Conclusion

By exploiting a combination of mass spectrometry, qRT-PCR, and cell culture assays, we demonstrated that MGL treatment of HT29 cancer cells caused cytotoxicity, chromatin and proteome regulation, and spurious transcription. Genomic instability was not a surprising phenomenon, as spurious transcription of DNA repetitive units was previously demonstrated by other authors (Mostoslavsky et al., 2006; Simon et al., 2019). Our work supports the notion that treatment with MGL of cancer cells may be useful in their growth inhibition due to its potential in affecting specific histone methylation marks and translation events. Future studies will have to unravel the global changes in spurious transcription and their mechanistic effects on cell death. It should also be pointed out that MGL-based cancer therapy may take advantage of selective and effective targeting of cancer cells via coupling strategies to specific surface recognition determinants.

## Data Availability

The data presented in the study are deposited in the Chorus repository (https://chorusproject.org/), accession number 1712.

## Abbreviations

BCA, bicinchoninic acid; DDA, data-dependent acquisition; DIA, data-independent acquisition mode; DMEM, Dulbecco’s Modified Eagle Medium; DTT, DL-dithiothreitol; FBS, fetal bovine serum; HO-Hxo-DH, D-2-hydroxyisocaproate dehydrogenase; MGL, methionine γ-lyase; NIB, Nuclei Isolation Buffer; PBS: Phosphate Buffered Saline; PLP, pyridoxal 5'-phosphate; PTMs, post-translational modifications; SAM, S-adenosylmethionine; SDC, sodium deoxycholate; TCA, trichloroacetic acid; TFA, trifluoroacetic acid.
