# Supplementary material for: A Key Silencing Histone Mark on Chromatin Is Lost When Colorectal Adenocarcinoma Cells Are Depleted of Methionine by Methionine γ-Lyase
Source: Front Mol Biosci. 2021 Oct 1;8:735303. doi: 10.3389/fmolb.2021.735303 (PMC8517235; doi:10.3389/fmolb.2021.735303)
Supplement: Supplementary file 1 [file Presentation1.pdf]

A KEY SILENCING HISTONE MARK ON CHROMATIN IS LOST WHEN COLORECTAL ADENOCARCINOMA CELLS ARE DEPLETED OF METHIONINE BY METHIONINE *γ*-LYASE

Samanta Raboni, Serena Montalbano, Stephanie Stransky, Benjamin A. Garcia, Annamaria Buschini, Stefano Bettati, Simone Sidoli and Andrea Mozzarelli

Supplementary figures

Supplementary Figure 1

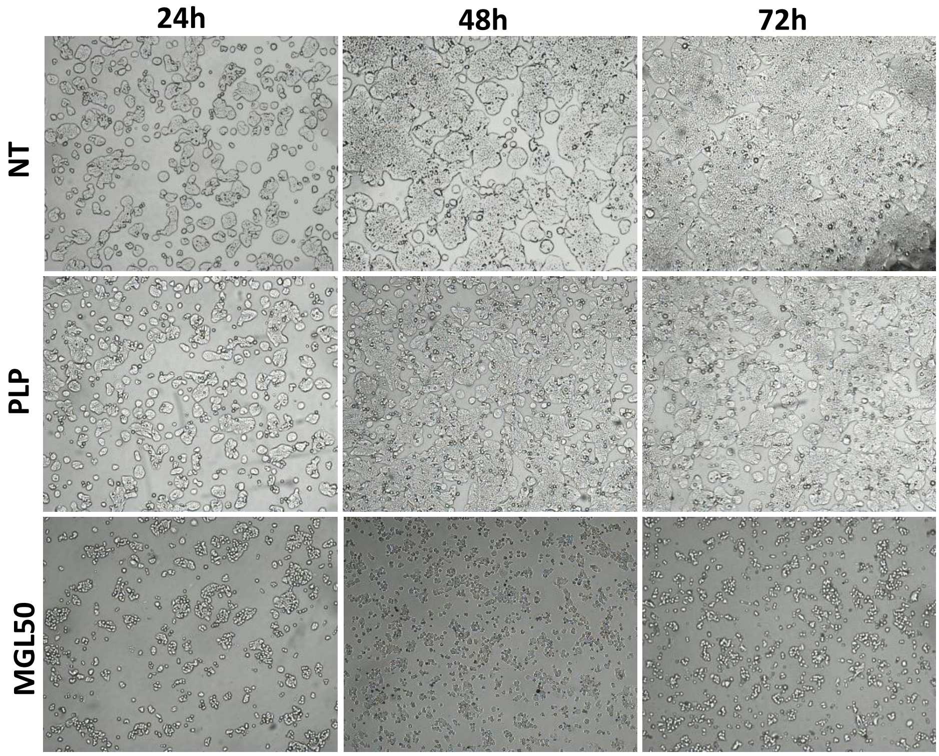

Figure S1. Microscopy images of HT29 cancer cells following treatment with MGL. HT29 cells were treated with PLP, MGL50 for 24, 48 and 72h. Control cells (NT) were kept in culture for the same period of time. Images were acquired using a JuLI Smart fluorescent cell analyzer instrument (Digital Bio Technology, Boston, USA). Magnification: 4 X, NA: 0.16

Supplementary Figure 2

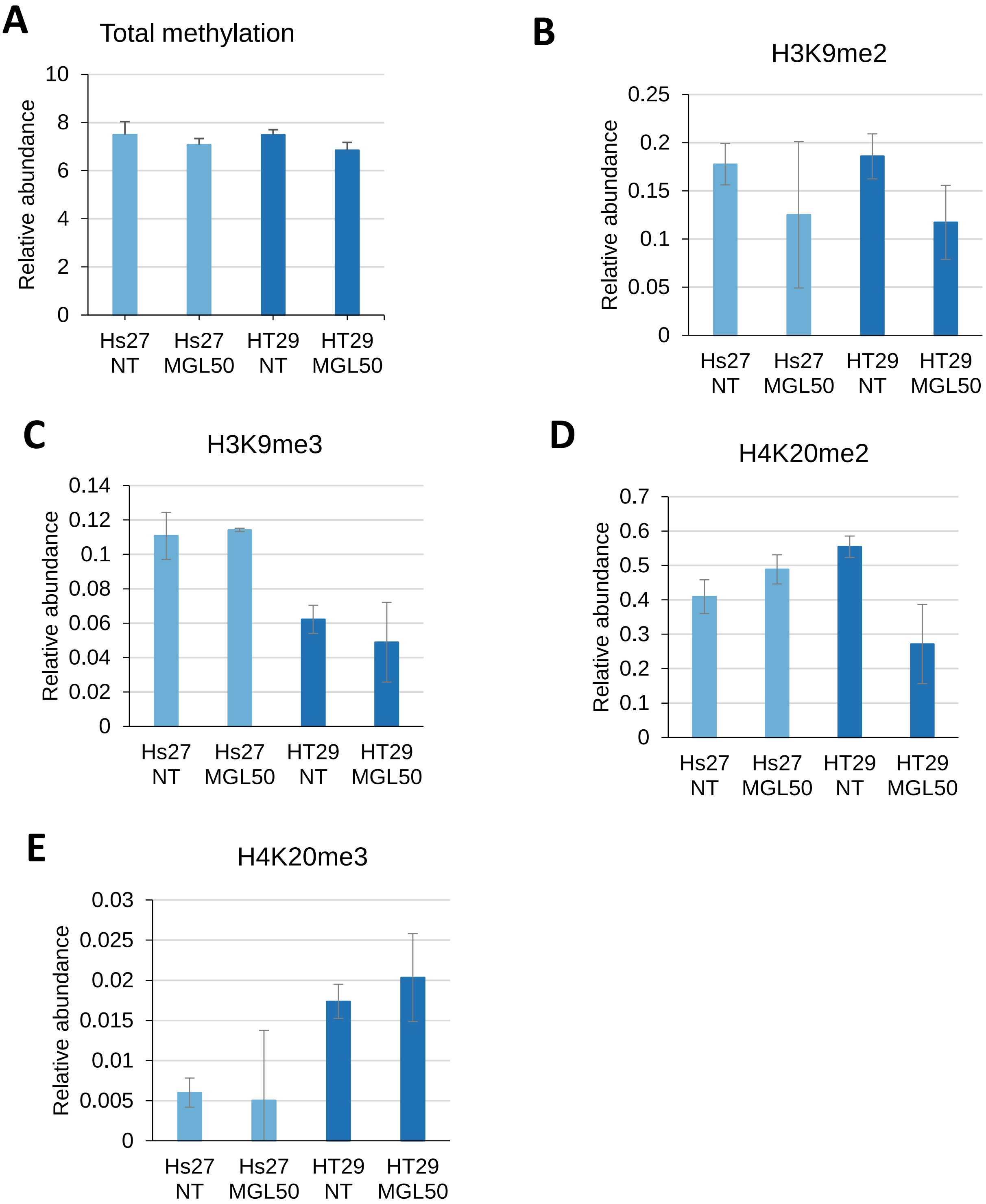

Figure S2. Relative abundance of selected histone methylations in Hs27 and HT29 cells (un)treated with MGL. (A) Relative abundance of total methylation level, calculated by summing the relative abundance of all peptides quantified by EpiProfile 2.0 containing a methyl mark. (B) Relative abundance of the total H3K9me2 mark, (C) the H3K9me3, (D) H4K20me2 and (E) H4K20me3.

Supplementary Figure 3

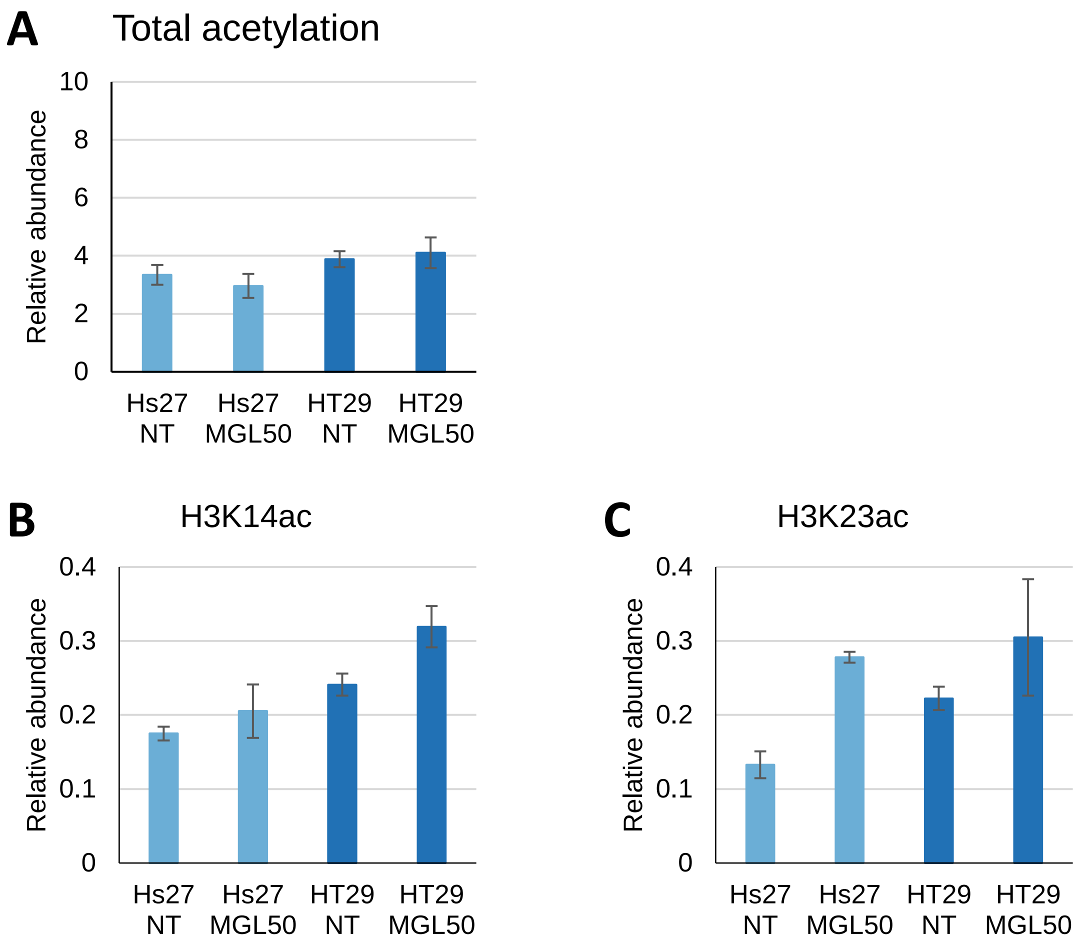

Figure S3. Relative abundance of selected histone acetylations in Hs27 and HT29 cells (un)treated with MGL. (A) Relative abundance of total acetylation level, calculated by summing the relative abundance of all peptides quantified by EpiProfile 2.0 containing an acetyl group. (B) Relative abundance of the total H3K14ac mark and (C) the H3K23ac.

Supplementary Figure 4

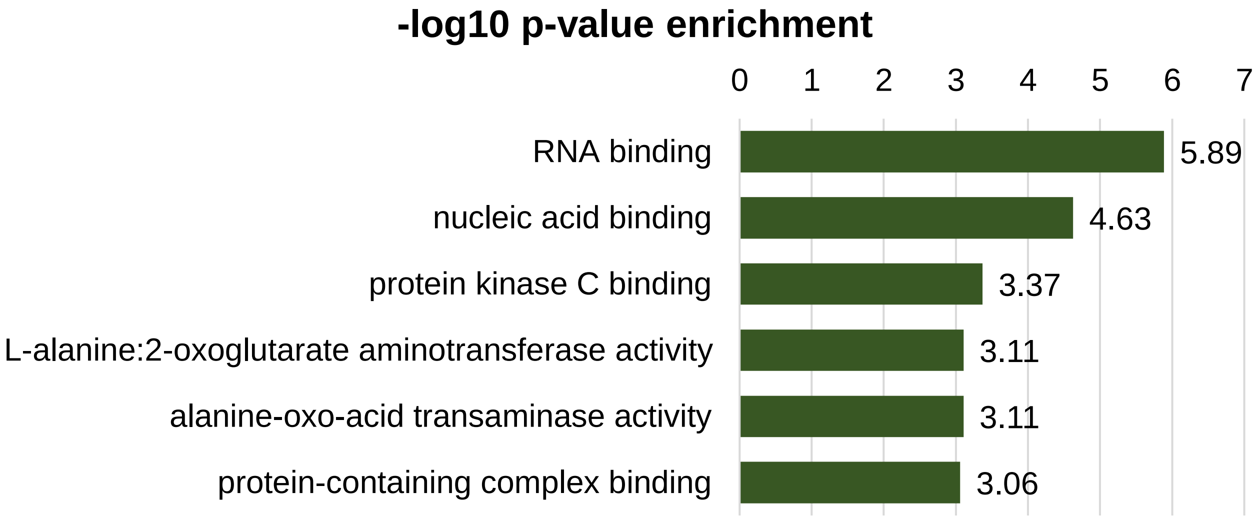

Figure S4. Enrichment of protein molecular function performed with the software GOrilla (PMID: 19192299). Proteins were sorted by the product fold change x p-value to prioritize those enriched in the PLP control, i.e. depleted by MGL treatment. Enrichment was calculated using the single ranked list algorithm, and data were displayed as negative Log10 of the calculated p-value.

Supplementary Figure 5

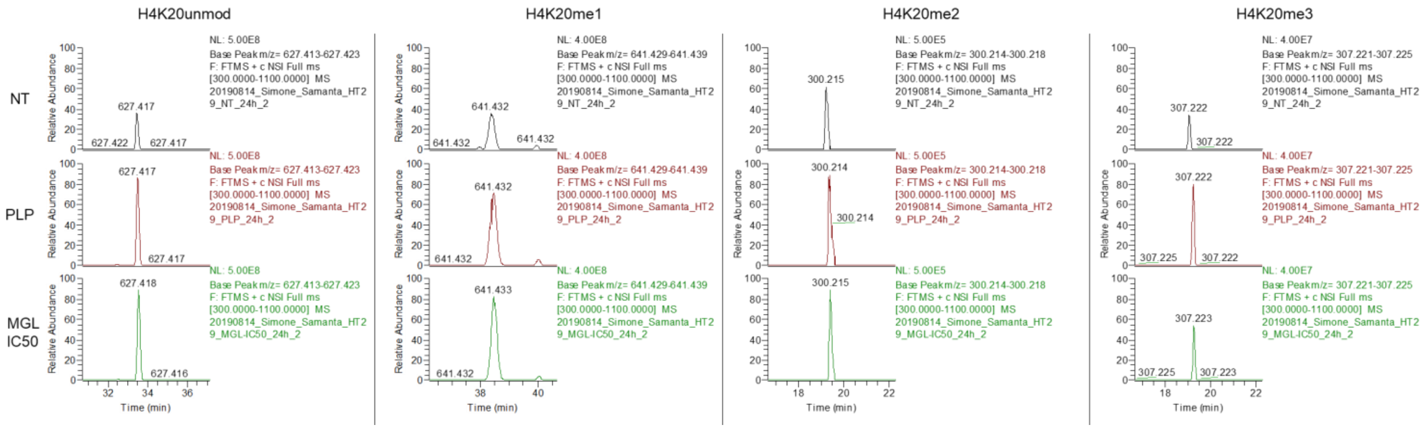

Figure S5. Extracted ion chromatogram of H4K20 modified peptides. Chromatographic signals for the unmod, me1, me2 and me3 peptide of the untreated sample (NT, top), PLP treated (middle) and MGL treated (bottom). Each peptide was scaled with the same signal height, so differences are more noticeable. MGL treatment shows the clearest decrease between the unmodified and the trimethyl form. The NT sample has signals for all peptides approximately comparable. H4K20me2 was also found to be significantly decreased in MGL treatment (Figure 2H), here confirmed by the narrower peak width compared to the other conditions.

Supplementary Figure 6

A

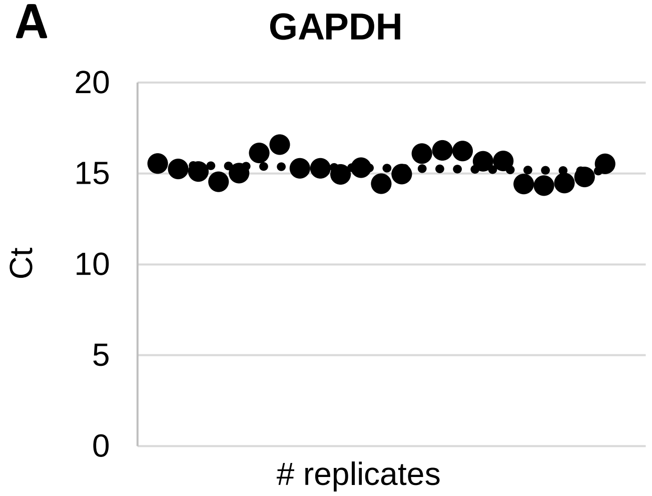

B

	GAPDH	
	PLP	MGL50
Ct	15.18 ± 0.20	15.87 ± 0.32

Figure S6. qRT-PCR GAPDH Ct variation. (A) Threshold Cycle (Ct) data of different experiments. (B) qRT-PCR data are expressed as threshold Cycle (Ct) mean ± standard deviation of GAPDH for the evaluation of Major Satellite expression.
